# Typhoid Fever and Unguentum Crede

**Published:** 1904-06

**Authors:** 


### Typhoid Fever and Unguentum Crede.

Leonard Weber, New York Post-Graduate Medical School and Hospital, Consulting Physician to Saint Mark's Hospital, New York, in a lecture on typhoid fever, delivered at the New York Post-Graduate Medical School, November 2, 1903, said: I have also the records of three cases of grave typhoid sepsis saved by inunctions of unguentum Credé—soluble metallic silver ointment rubbed in thoroughly over various parts of the body, in doses of ½ dram. Though the usual stimulants and excitants were also made use of in these cases, I believe, nevertheless, that they were really saved by the antiseptic action of the Credé preparation.

